# Thyroid function tests and resting heart rate in Graves’ disease: a prospective longitudinal study

**DOI:** 10.1530/EC-26-0129

**Published:** 2026-06-12

**Authors:** Beatrice Egger, Uwe Riedmann, Lisa Schmitt, Daniel Arian Kraus, Hannah Pichler, Christian Trummer, Verena Theiler-Schwetz, Stefanie Lindschinger, Winfried März, Oleksiy Tsybrovskyy, Markus Reichhartinger, Stefan Pilz

**Affiliations:** ^1^ Department of Internal Medicine, Hospital of The Brothers of St. John of God, Graz, Austria; ^2^ Department of Internal Medicine, Division of Endocrinology and Diabetology, Medical University of Graz, Graz, Austria; ^3^ Synlab Academy, Synlab Holding GmbH, Mannheim, Germany; ^4^ Diagnostic and Research Institute of Pathology, Medical University of Graz, Graz, Austria; ^5^ Institute of Automation and Control, Graz University of Technology, Graz, Austria

**Keywords:** hyperthyroidism, thyrotoxicosis, wearables, Fitbit, monitoring, smartwatch

## Abstract

**Objective:**

Increased resting heart rate (RHR) is a typical sign of thyrotoxicosis. RHR may therefore serve as a monitoring tool, but longitudinal data on continuous measures of RHR and thyroid hormones are sparse. We evaluated the association between continuously monitored RHR and thyroid function tests in Graves’ disease (GD).

**Design:**

This is a double-center, prospective, longitudinal cohort study in 30 patients with GD on antithyroid drug treatment. Participants were followed up with monthly study visits over six months at two hospitals in Graz, Austria.

**Methods:**

Free thyroxine (fT4) and free triiodothyronine (fT3) were measured at each study visit. Participants were encouraged to continuously wear a wristband activity tracker (Fitbit Charge 6) to assess RHR. Associations of RHR (mean value over ten days) with thyroid hormones were determined by generalized estimating equation analyses.

**Results:**

From September 2024 to November 2025, we enrolled 30 participants (24 women, mean (SD) age of 40.7 (14.6) years) and performed 176 study visits. Thyrotoxicosis was present in 24 participants (80%) at baseline. One SD increase in RHR of 8.8 beats per minute was associated with fT4 (beta = 0.856, 95% CI: 0.641–1.071, *P* < 0.001), fT3 (beta = 0.836, 95% CI: 0.620–1.051, *P* < 0.001), and thyrotoxicosis (odds ratio: 5.134, 95% CI: 2.192–12.025, *P* < 0.001), respectively.

**Conclusion:**

RHR assessed by a wearable device is strongly associated with thyroid hormones in GD. Further trials are required to evaluate whether RHR is useful to guide timing of thyroid function tests during antithyroid drug treatment and for GD diagnostics.

## Introduction

Graves’ disease (GD) is the most common cause of hyperthyroidism, i.e. increased thyroid hormone synthesis and secretion resulting in increased circulating thyroid hormones termed thyrotoxicosis (1, 2, 3, 4). GD affects approximately 0.5–1.0% of the population and is an autoimmune disease characterized by autoantibodies against the thyroid-stimulating hormone (TSH) receptor (TRAbs) (1, 2, 3, 4). There are three first-line therapeutic options for the treatment of GD, i.e. antithyroid drug (ATD) treatment, radioactive iodine (RAI) therapy, and thyroid surgery (1, 2, 3). ATDs, mainly methimazole (MMI), are nowadays the preferred first-line therapy worldwide with a traditional treatment duration of 12–18 months and a recent increase in the use of long-term ATD (LTATD) therapy ≥ 2 years due to improved outcomes with this approach (1, 4). Dose titration and surveillance of ATD therapy require regular check-ups for the determination of thyroid hormones that are relatively costly with usually heterogeneous and arbitrary timelines between repeated testing as there is no clear management guidance (1, 2, 3, 4, 5, 6).

Elevated resting heart rate (RHR) is a typical sign of thyrotoxicosis, and thyroid hormones are significantly associated with RHR even in populations with largely normal thyroid function, although data are largely restricted to less useful single measures of RHR instead of more accurate continuous measurements of RHR over several days (1, 3, 7, 8, 9). RHR may thus be a promising tool for monitoring patients with GD on ATD to guide timing of follow-up examinations, aid in ATD dose titration, and improve detection of recurrence after ATD discontinuation (1, 7, 8, 9, 10, 11, 12, 13, 14). Although the concept of using RHR in the clinical management of GD seems obvious, data from prospective longitudinal studies on this issue are sparse and insufficient for wide implementation of RHR in clinical routine care, thus requiring further investigations (1, 10, 11, 12, 13, 15).

In this prospective longitudinal study in patients with GD, we performed thyroid function tests, i.e. measurements of free thyroxine (fT4), free triiodothyronine (fT3), and TSH, monthly for 6 months and continuously measured RHR with a wristband activity tracker (Fitbit Charge 6) (16). Primary outcome analyses were the associations between thyroid hormones and RHR.

## Materials and methods

This is a double-center, prospective, longitudinal cohort study in 30 patients with GD on ATD treatment termed ‘THYroid hormones and resting HEART rate’ (THYHEART) pilot study. Study participants were recruited from the outpatient clinics of the Department of Internal Medicine, Division of Endocrinology and Metabolism, Medical University of Graz (MUG), and the Department of Internal Medicine, Hospital of the Brothers of St. John of God, Graz, Austria.

We enrolled consecutive adult patients (aged ≥ 18 years) with GD diagnosed according to the respective guidelines of the European Thyroid Association (ETA) who were routinely referred to our departments (2). Patients were eligible for study inclusion if ATDs were taken for less than 30 days or more than one year with, according to the treating physician, likely discontinuation of ATD therapy within the study period of 6 months. The rationale behind this was to include patients with likely changing thyroid function tests during the study period, which usually occurs after starting ATD therapy, and with potential disease recurrence after ATD discontinuation. Pregnancy was an exclusion criterion. No formal sample size calculation was performed for this pilot study, although the sample size of 30 seems sufficient in view of the only comparable study in this field (12, 13). This study received no external funding or any company support. We obtained approval by the local ethics committees at both study centers, and all study participants gave written informed consent. This publication adheres to the Strengthening the Reporting of Observational Studies in Epidemiology (STROBE) statement guidelines and complies with Good Clinical Practice and the Declaration of Helsinki (17).

On the day of study inclusion, the first study visit (study visit 1) was performed followed by further study visits with monthly intervals for 6 months (study visits 2–7) and study end either at the 7th study visit or if 7 months elapsed after study inclusion. At each study visit, venous fasting blood samplings were performed between 07:30 and 12:00 h. fT4, fT3, and TSH were determined by electrochemiluminescence immunoassay (ECLIA) (Roche® Cobas, Germany, at both study centers according to manufacturer’s instructions), and body weight (to the nearest 0.1 kg) and height (to the nearest 0.1 cm) were measured at study visit 1 with the participants wearing no shoes and only light clothes to calculate body mass index (BMI) as weight (kg)/(height (m))^2^. Systolic and diastolic blood pressure (BP) (in mmHg) were determined by automated routine devices after 5 min in a sitting position and also included a measurement of heart rate (in beats per minute; bpm) (termed on-site-RHR). Current medication use of ATD and beta-blockers (drug name and daily dosage at study visit day) was recorded at all study visits along with assessment of clinical symptoms.

At study inclusion, all participants received a wristband activity tracker, Fitbit Charge 6 (Fitbit, USA), and were encouraged to continuously wear this device as much as possible, even when sleeping (16). They received instructions for the device and the use of the Fitbit apps on Android or iOS. Account information for the Fitbit app was newly generated for the purpose of the study and shared with the researchers to have access to the study-related data. RHR by Fitbit Charge 6 is measured by photoplethysmography with acceptable accuracy in validation studies (16). RHR is provided by Fitbit based on HR and activity data from the device by use of an undisclosed algorithm that was well aligned with an algorithm using HR data for time windows with the absence of tracked physical activity for 15 min or more (12, 16). The primary outcome measure was the mean RHR over 10 days before the study visit (termed RHR-Fitbit-10d), with the exception of the first study visit for which we recorded the 10 days after the study visit. The rationale behind the 10-day period is that it may be less affected by day-to-day variations and that one previous study showed stronger associations with thyroid hormones compared with shorter or longer time periods (12, 13, 18). Accordingly, we also calculated RHR-Fitbit-5d, RHR-Fitbit-20d, and RHR just on the day of the study visit (RHR-study visit), as additional outcome measures. All RHR values were considered eligible (valid) for analyses if the RHR of at least one day was recorded. No data imputation for any missing value was performed.

At each study visit, we also determined TRAbs and pre-specified secondary outcome measures of this study, i.e. the following German language questionnaires: ‘Hyperthyroid Symptom Scale’, ‘THyPro39de’, and the ‘psychosomatic assessment health disc (PAHD)’ (19, 20, 21). Analyses of these secondary outcomes will be published in separate articles.

Depending on the data distribution, as evaluated by descriptive statistics, continuous data are either shown as mean ± standard deviation (SD) or as median with interquartile range (IQR), whereas categorical data are shown as percentages. Baseline (at study visit 1) clinical and laboratory characteristics are shown for the entire study cohort and stratified according to study center and according to the presence or absence of thyrotoxicosis (i.e. overt or clinical hyperthyroidism) defined as fT4 and/or fT3 concentrations above the respective reference range. Group comparisons are done by Student’s *t* test, Mann–Whitney U test, or chi-square test, as appropriate. The association between thyroid function tests (dependent variable) and RHR measures (independent variable) was determined by linear model generalized estimating equation (GEE) analyses (22, 23). We used binary logistic model GEE analyses to calculate odds ratios (ORs) (95% CIs) for thyrotoxicosis per one SD increase in RHR measures. Pre-specified subgroup analyses were planned according to sex (women/men), use of beta-blockers at the respective study visit (yes/no), thyrotoxicosis (yes/no), and study center. According to data suggesting that beta-blockers may decrease RHR by about 11 bpm, we re-calculated our analyses by adding 11 bpm to all RHR measures assessed during concomitant intake of beta-blockers at the respective study visit (24). The exception was the first study visit for which beta-blocker use was classified according to the prescription at this study visit for RHR measures assessed thereafter (24). For descriptive purposes, we calculate, for each individual, the mean RHR-Fitbit-10d values at study visits with normal thyroid function (fT4, fT3, and TSH within the reference range) and thyrotoxicosis and the respective numerical and percentages differences in RHR measures. All statistical analyses were pre-specified and were conducted using R (R statistical software, version 4.4.2, R Foundation for Statistical Computing, Austria). A *P*-value <0.05 was considered statistically significant.

## Results

This study was conducted from September 26, 2024, to November 7, 2025. After screening 31 consecutive patients, we enrolled 30 study participants. The baseline characteristics of all study participants are shown in Table 1. During follow-up, two study participants dropped out before the second visit: one dropped out due to pregnancy and one was lost to follow-up. Of the remaining 28 participants, 17 completed all 7 study visits and 7 completed 6 study visits before study end (Table S1 (see section on Supplementary materials given at the end of the article)). Overall, thyroid hormone data from 176 study visits were available for analyses with a median (IQR) interval between study visits of 29 (28–35) days (Table 1). RHR-Fitbit-10d was based on daily RHR data of all respective ten days in 72% of the available measures. We could not analyze RHR data of two participants due to technical problems with online data access. Some additional missing RHR data were due to partially incomplete adherence to wearing the Fitbit device, thus explaining the different sample sizes for some of the analyses. Concomitant beta-blocker use was recorded at 60 (34%) of the study visits. Methimazole (MMI) was the preferred ATD and used by 29 participants at least once, with a mean (SD) daily dose of 10.9 (10.5) mg at 150 study visits. Five participants discontinued MMI during the follow-up period with no GD disease recurrence, i.e. no thyrotoxicosis, until study end.

**Table 1:** Baseline characteristics at study visit 1.

Variable	All	MUG	St. John	*P*-value MUG vs St. John
Number	30	15	15	
Females (%)	24 (80.0%)	15 (100.0%)	9 (60.0%)	0.022
Age (years)	40.7 (14.6)	40.2 (14.6)	41.3 (15.0)	0.967
Weight (kg)	70.6 (9.9)	69.4 (7.8)	71.8 (11.6)	0.662
Height (cm)	167.7 (8.8)	164.0 (6.0)	171.7 (9.7)	0.027
BMI (kg/m^2^)	25.0 (3.5)	25.9 (3.4)	23.9 (3.5)	0.128
fT4 (pmol/L) (reference range: 11.9–21.6)	34.8 (20.7)	38.6 (24.7)	31.0 (15.7)	0.619
fT3 (pmol/L) (reference range: 3.1–6.8)	14.9 (10.0)	15.7 (11.4)	14.1 (8.6)	0.917
TSH (μIU/mL) (reference range: 0.27–4.20)	0.14 (0.39)	0.22 (0.53)	0.05 (0.11)	0.609
Thyrotoxicosis (%)	24 (80.0%)	11 (73.3%)	13 (86.7%)	0.648
Systolic BP (mmHg)	136.6 (18.0)	131.4 (14.4)	141.8 (20.2)	0.177
Diastolic BP (mmHg)	79.4 (11.0)	79.5 (11.5)	79.3 (10.8)	0.950
On-site-RHR (bpm)	88.6 (17.8)	88.8 (12.5)	88.4 (22.4)	0.493
RHR-Fitbit-5d (bpm)	76.8 (10.3)	77.7 (10.4)	76.0 (10.5)	0.691
RHR-Fitbit-10d (bpm)	77.1 (9.9)	78.0 (9.5)	76.2 (10.6)	0.550
RHR-Fitbit-20d (bpm)	75.8 (9.3)	76.6 (7.9)	75.1 (10.8)	0.488
Beta-blockers (%)	9 (30.0%)	4 (26.7%)	5 (33.3%)	1.000
ATD (%)	20 (66.7%)	9 (60.0%)	11 (73.3%)	0.699

Values are expressed as mean (standard deviation) for continuous variables and as percentages for categorical variables.

*P*-values are calculated by Student’s *t*-test, Mann–Whitney U test, or chi-square test as appropriate.

Note that RHR-Fitbit values are based on measures after the visit, as they pertain to visit 1.

BMI, body mass index; fT4, free thyroxine; fT3, free triiodothyronine; TSH, thyroid-stimulating hormone; BP, blood pressure; RHR, resting heart rate; bpm, beats per minute; ATD, antithyroid drug; MUG, Medical University of Graz.

**Figure 1:**
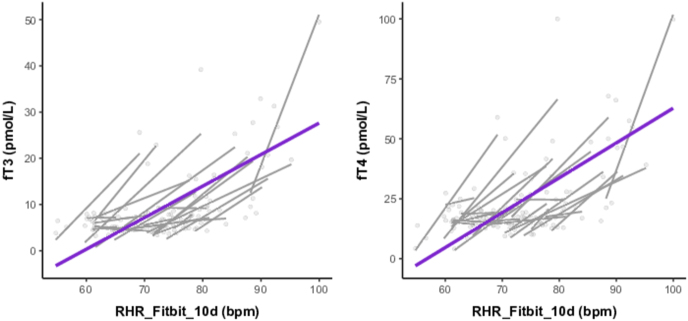
Association between RHR-Fitbit-10d (in beats per minute; bpm) and hormone measures of fT3 (left) and fT4 (right). The main association shown in purple represents the GEE-estimated association, while the gray lines show linear regression of individuals.

The results from our primary outcome analyses on the association of RHR measures with thyroid function tests in the entire study cohort are shown in Tables 2 and 3. In brief, there were significant positive associations between RHR measures and fT4 and fT3, while the associations with TSH were only marginally significant. The OR (95% CI) for thyrotoxicosis for one SD in RHR-Fitbit-10d (i.e. 8.8 bpm) was 5.134 (2.192–12.025; *P* < 0.001). The GEE analyses of fT3 and fT4 are graphically displayed in Fig. 1. Sensitivity analyses and analyses adding 11 bpm to RHR data assessed during concomitant use of beta-blockers revealed similar results, except for no significant association of visits in which participants were euthyroid (Table S2, S3, S4, S5). Regarding beta-blockers, we show the drug names and daily dosages at each visit in Table S6.

**Table 2:** Linear model generalized estimating equation (GEE) analyses for associations between thyroid function tests and measures of resting heart rate (RHR).

	*n*	Unstandardized beta (95% CI)	Standardized beta (95% CI)	*P*-value
**Free thyroxine (fT4)**				
On-site-RHR	167	0.575 (0.324, 0.826)	0.564 (0.318, 0.810)	<0.001
RHR-Fitbit-5d	131	1.331 (0.964, 1.697)	0.804 (0.583, 1.026)	<0.001
RHR-Fitbit-10d	139	1.455 (1.090, 1.820)	0.856 (0.641, 1.071)	<0.001
RHR-Fitbit-20d	143	1.434 (1.055, 1.813)	0.802 (0.590, 1.014)	<0.001
RHR-Fitbit-study visit	126	1.364 (0.776, 1.953)	0.742 (0.422, 1.062)	<0.001
**Free triiodothyronine (fT3)**				
On-site-RHR	167	0.254 (0.137, 0.371)	0.509 (0.274, 0.744)	<0.001
RHR-Fitbit-5d	131	0.637 (0.465, 0.808)	0.810 (0.592, 1.028)	<0.001
RHR-Fitbit-10d	139	0.683 (0.507, 0.860)	0.836 (0.620, 1.051)	<0.001
RHR-Fitbit-20d	143	0.669 (0.475, 0.864)	0.766 (0.543, 0.989)	<0.001
RHR-Fitbit-study visit	126	0.650 (0.344, 0.957)	0.730 (0.385, 1.074)	<0.001
**Thyroid-stimulating hormone (TSH)**				
On-site-RHR	167	−0.024 (−0.047, −0.000)	−0.299 (−0.592, −0.005)	0.046
RHR-Fitbit-5d	131	−0.035 (−0.067, −0.003)	−0.308 (−0.591, −0.025)	0.033
RHR-Fitbit-10d	139	−0.035 (−0.068, −0.003)	−0.313 (−0.597, −0.028)	0.031
RHR-Fitbit-20d	143	−0.030 (−0.052, −0.007)	−0.279 (−0.490, −0.068)	0.010
RHR-Fitbit-study visit	126	−0.026 (−0.046, −0.007)	−0.249 (−0.433, −0.066)	0.008

**Table 3:** Binary logistic model generalized estimating equation (GEE) analyses for associations between thyrotoxicosis and measures of resting heart rate (RHR).

	*n*	Odds ratio (95% CI)	Odds ratio per SD RHR increase (95% CI)	*P*-value
On-site-RHR	167	1.071 (1.036, 1.106)	3.160 (1.821, 5.483)	<0.001
RHR-Fitbit-5d	131	1.185 (1.090, 1.289)	4.591 (2.160, 9.759)	<0.001
RHR-Fitbit-10d	139	1.204 (1.093, 1.326)	5.134 (2.192, 12.025)	<0.001
RHR-Fitbit-20d	143	1.215 (1.091, 1.354)	6.156 (2.250, 16.843)	<0.001
RHR-Fitbit-study visit	126	1.173 (1.089, 1.264)	4.733 (2.292, 9.775)	<0.001

Thyrotoxicosis is defined as fT3 and/or fT4 above the reference range.

Descriptive RHR-Fitbit-10d data for each individual according to thyroid function are shown in Table 4. The numerical mean difference in RHR-Fitbit-10d between normal thyroid function and thyrotoxicosis was 7.2 bpm and ranged from −1.2 to 19.2 bpm.

**Table 4:** Mean resting heart rate (RHR-Fitbit-10d) values of each participant at visits with thyrotoxicosis and normal thyroid function.

Study participant	Thyrotoxicosis (A)	Normal thyroid function (B)	A – B (% of B)
1	84	72.2	11.8 (16.4%)
2			
3	89.7	78.7	11 (14%)
4	82.6	73.9	8.7 (11.8%)
5	63.2	55.5	7.7 (13.9%)
6	74.3	72.2	2.1 (2.9%)
7	68.5	61.4	7 (11.4%)
8	70.8	61.1	9.8 (16%)
9		65.8	
10	79.6	80.8	−1.2 (−1.4%)
11	69.1	67.6	1.4 (2.1%)
12	65.5		
13	62.1	62	0.1 (0.1%)
14	89.9		
15	77.5		
16	79.6	72.9	6.7 (9.1%)
17		62.7	
18	78.1	75.5	2.7 (3.6%)
19		70	
20	76.4	69.1	7.3 (10.5%)
21		67.1	
22	77.6	73.7	3.9 (5.3%)
23	79.2	64.1	15 (23.5%)
24			
25			
26	78.3		
27	77.8	73	4.8 (6.5%)
28	87.1	76.1	11.1 (14.5%)
29	85.5	66.3	19.2 (28.9%)
30	93.3		

## Discussion

In this double-center, prospective longitudinal study in patients with GD, we documented significant positive associations between thyroid hormones and RHR assessed by the wristband activity tracker Fitbit Charge 6.

Our results confirm the findings of the methodological benchmark studies on RHR measured by wristband activity trackers and thyroid hormones by Shin *et al.* and Lee *et al.* (11, 12, 25). One of these studies from Korea by Lee *et al.* included 30 patients with thyrotoxicosis (mainly but not exclusively due to GD) and ten controls and used Fitbit Charge 2 to assess RHR (12, 13). Lee *et al.* did not include fT3 data, had a shorter follow-up time than our study (i.e. 3 versus 6 months of monthly visits), and used the mean RHR of the five days before the thyroid function tests as the primary outcome measure (12, 13). The associations between RHR and thyroid hormones were even stronger in our study as compared to Lee *et al.* This may be partially attributed to differences in participant characteristics, such as intra-individual variations in thyroid hormone concentrations or different ethnicities, and to the fact that we used RHR-Fitbit-10d instead of RHR-Fitbit-5d as our primary outcome measure. In line with another previous investigation by Shin *et al.* in 175 patients with various forms of thyroid dysfunctions, RHR-Fitbit-10d seems to be more strongly associated with thyroid hormones than RHR-Fitbit-5d (see Tables 2 and 3) (11). Thus, our findings are a confirmatory extension of the study by Shin *et al.* (11). As another confirmation of the studies from Korea, RHR-Fitbit-10d was clearly a superior predictor of thyroid hormones compared with on-site-RHR, suggesting that assessments of RHR during an appointment at an outpatient clinic are of limited diagnostic utility compared to average continuous at-home measures, such as RHR-Fitbit-10d (11, 12, 13). This underscores the significance of our work using a wristband activity tracker to continuously assess RHR, while the vast majority of previous studies on RHR and thyroid hormones used different kinds of on-site-RHR measures (7, 8, 26). Of note, TSH is usually suppressed in thyrotoxicosis with low variance and is thus a limited parameter for capturing differences in thyroid function in this setting. Dynamic uncoupling of TSH from peripheral thyroid hormones during ATD dose titration and limited variability in subclinical ranges may also play a role. This may explain why TSH was often only borderline significantly associated with RHR in our analyses. Therefore, we do not consider differences in some sensitivity analyses (e.g. between study centers) regarding associations of TSH with RHR as relevant, but wish to stress that most results were similar.

Although MMI is frequently prescribed with the beta-blocker propranolol that decreases heart rate, our results remained significant even when restricting the analyses to users of beta-blockers (13, 15, 24). It is important to note that propranolol has a relatively short half-life of 3–4 h (maximum effect after approximately 60–90 min) and is often only taken on demand and not continuously, while RHR is permanently monitored, thus potentially explaining a rather minor effect of propranolol on RHR data in GD (13, 15). Importantly, adjusting RHR values in users of beta-blockers by adding 11 bpm revealed similar results as without adjustments, although we acknowledge that these analyses may be considered over-adjustments due to the above-mentioned reasons (24). In this context, we wish to point out that use of beta-blockers is very rare after achieving euthyroidism under ATD therapy or after withdrawal of ATD therapy. Thus, the potential impact of beta-blockers may be negligible when using RHR measure for detection of GD recurrence or during long-term ATD therapy monitoring, which are promising fields for its potential use in the future.

From a pathophysiological point of view, it is well established that thyroid hormones exert several effects on the cardiovascular system, including an increase in RHR (7, 26, 27). Precise molecular pathways for the chronotropic effect of thyroid hormones are largely unclear, but evidence suggests that this effect is rather mediated by direct effects on the heart than by indirect effects via the brain and subsequent impact on the autonomic nervous system (7). Large epidemiological studies in the general population showed that there is an increased cardiovascular risk in patients with higher thyroid hormone levels (26, 28). As RHR itself is also a risk factor for cardiovascular disease and mortality, it is tempting to hypothesize that the chronotropic effects of thyroid hormones might also be harmful regarding hard clinical outcomes (29, 30, 31, 32). In general, increases in RHR might be useful for the detection of all sorts of hyperthyroidism and thyrotoxicosis (e.g. by overdosing of thyroid hormone replacement therapy). Therefore, our findings together with the existing literature may inform future trials aiming to implement RHR measures as a routine diagnostic tool in the management of thyroid dysfunction to improve clinical outcomes (1, 10). The enormous potential of this approach is underlined by data from adults in the US from 2022 reporting on a wearable device adoption of 36.4%, with 78.4% of these individuals being willing to share their data with healthcare providers, which is consistent with data from other countries (33, 34).

Our findings provide estimates on the effect sizes of RHR increases that are indicative of thyrotoxicosis. For example, beyond our GEE analysis results, the simple numerical difference in RHR between normal thyroid function and thyrotoxicosis with a mean increase of 7.2 bpm (range: from −1.2 to 19.2 bpm) provides at least a rough estimate for clinicians and patients on when to suspect, e.g. GD recurrence. The relatively wide range of differences in RHR between normal thyroid function and thyrotoxicosis points to the need for personalized, baseline-anchored *ΔRHR* approaches for clinical applications. It is tempting to hypothesize that if RHR was continuously tracked by a wearable device following the diagnosis of GD, differences in RHR may provide an individual personalized indicator for potential disease recurrence of GD during long-term follow-up. While replacement of thyroid hormone function tests by RHR measures is currently not viable, there is a general clinical need to improve guidance for timing of follow-up laboratory measurements in GD patients on ATD treatment and their surveillance after ATD discontinuation. Guidelines on this issue do not provide clear guidance with, for example, recommendations for follow-up thyroid function tests ranging from 2 weeks to 6 months (1, 6). A common practice in Austria is monthly follow-up visits for GD patients on ATD treatment that are costly, resource demanding, and of questionable cost-effectiveness at this high frequency (1, 2, 5). Our findings may hopefully inform randomized trials comparing less frequent testing guided by RHR measures compared with a usual care group to assess the clinical utility of RHR-based surveillance in GD patients. These future trials may also provide data for incorporation of RHR measures in decision support systems and artificial intelligence (AI) tools for dose titration of ATD therapy and general management of thyroid dysfunctions (10, 11, 35). As wearable device technology is currently advancing, it is conceivable that in the future, RHR data may be combined with other measures related to thyroid hormone effects, such as body temperature, sweating, or tremor, to further improve diagnostic accuracy in clinical care of GD.

Our study is limited by a relatively low sample size and participants recruited from specialized departments for thyroid diseases, which limits the generalizability of our findings. In addition, we acknowledge that the algorithm for calculating RHR by Fitbit is undisclosed and that wrist-based devices for assessment of RHR are limited compared with chest strap-based monitors, although this potential limitation may be less relevant when analyzing intra-individual changes in RHR as in our study. In general, Fitbit Charge 6 has an acceptable accuracy and the advantages of a high popularity and easy accessibility (16). Another limitation is that we did not systematically record all factors with potential relevance for RHR (e.g. illness, extraordinary emotional states, and actual beta-blocker intake for every day). Uniform adjustment with +11 bpm for beta-blocker use does not capture the heterogeneity of beta-blockers and exposure timing relative to the 10-day averaging window. The main strengths are that this is the first study in GD patients reporting on associations of fT3 with RHR assessed by a wristband activity tracker, the first in this field in Caucasians, and the one with the longest individual follow-up time. Findings were similar at both study centers despite some differences in population characteristics, such as gender imbalance. In general, our work confirms previous studies from South Korea using more advanced devices in a different ethnicity, thus increasing the generalizability of wearable-based thyroid function monitoring solutions (11, 12). Associations of comparable magnitude were observed across earlier Fitbit Charge models and the current Fitbit Charge 6, supporting the robustness of the findings across device generations (11, 12, 13).

In conclusion, in a cohort of GD patients, we observed a significant association between thyroid hormone function tests and RHR measured using a wristband activity tracker (Fitbit Charge 6). This work replicates associations previously established in East Asian cohorts in a Caucasian population, supporting the cross-ethnic generalizability of wearable-based thyroid monitoring and the robustness of findings across device generations. Our findings provide a useful basis for further investigations on the use of RHR measures in the care of individuals with thyroid dysfunctions.

## Supplementary materials



## Declaration of interest

The authors declare that there is no conflict of interest that could be perceived as prejudicing the impartiality of the work reported.

## Funding

This research did not receive any specific grant from any funding agency in the public, commercial, or not-for-profit sector.

## Data availability

The data are available upon reasonable request and approval by our ethics committees.

## Ethics statement

This study was approved by the Ethics Committee at the Medical University of Graz.
