# Supplementary material for: Thyroid function tests and resting heart rate in Graves’ disease: a prospective longitudinal study
Source: Endocr Connect. 2026 Jun 12;15(6):e260129. doi: 10.1530/EC-26-0129 (PMC13266306; doi:10.1530/EC-26-0129)
Supplement: Supplementary file 1 [file supplementary_materials.pdf]

SUPPLEMENT: Thyroid function tests and resting heart rate in Graves` disease: a prospective longitudinal study

Table S1: Values per visit

Parameter	Visit 1	Visit 2	Visit 3	Visit 4	Visit 5	Visit 6	Visit 7
Thyrotoxicosis group, n	30	28	27	26	24	24	17
fT3 (pmol/L), mean (SD)	14.93 (9.98), n=30	7.86 (4.73), n=28	7.61 (8.58), n=27	8.01 (9.02), n=26	5.99 (1.66), n=24	7.87 (9.44), n=24	7.48 (6.94), n=17
fT4 (pmol/L), mean (SD)	34.78 (20.70), n=30	21.16 (10.60), n=28	19.12 (17.05), n=27	20.85 (18.48), n=26	16.97 (6.01), n=24	21.51 (18.50), n=24	20.60 (14.44), n=17
TSH (mIU/L), median (IQR)	0.01 (0.00), n=30	0.01 (0.05), n=28	0.04 (0.36), n=27	0.02 (0.97), n=26	0.12 (1.52), n=24	0.09 (0.79), n=24	0.15 (0.59), n=17
On-site RHR (bpm), mean (SD)	88.60 (17.84), n=30	79.83 (17.07), n=24	78.85 (18.90), n=27	79.04 (12.73), n=24	76.67 (12.61), n=24	79.96 (18.74), n=23	80.00 (16.74), n=17
RHR-Fitbit-5d (bpm), mean (SD)	76.79 (10.27), n=24	72.82 (10.13), n=24	69.50 (8.00), n=21	71.98 (10.01), n=19	70.66 (7.06), n=19	70.23 (6.71), n=18	71.89 (5.29), n=7
RHR-Fitbit-10d (bpm), mean (SD)	77.07 (9.94), n=27	73.01 (10.00), n=25	70.32 (7.58), n=22	71.58 (10.04), n=19	70.58 (6.78), n=19	70.37 (6.86), n=18	72.82 (5.64), n=10
RHR-Fitbit-20d (bpm), mean (SD)	75.79 (9.34), n=27	73.92 (9.41), n=25	70.45 (7.54), n=22	71.69 (9.65), n=20	70.86 (7.41), n=19	72.09 (9.92), n=20	75.56 (12.61), n=11
RHR-Fitbit-study visit (bpm), mean (SD)	78.52 (10.29), n=20	71.69 (9.21), n=23	70.33 (10.63), n=21	71.42 (9.97), n=19	70.76 (7.23), n=19	72.17 (10.71), n=18	71.94 (4.59), n=7

Abbreviations: fT4, free thyroxine; fT3, free triiodothyronine; TSH, thyroid-stimulating hormone; RHR, resting heart rate, bpm; beats per minute

Table S2. Stratified analyses of linear model generalized estimating equation (GEE) analyses for associations between thyroid function tests and RHR-Fitbit-10d.

Category	Hormone	n	Unstandardized b (95% CI)	Standardized beta (95% CI)	P-value
**Sex**					
female	fT4	111	1.479 (1.064 - 1.894)	0.792 (0.570 - 1.014)	<.001
female	fT3	111	0.717 (0.500 - 0.933)	0.790 (0.552 - 1.028)	<.001
female	TSH	111	-0.047 (-0.085 - -0.008)	-0.362 (-0.661 - -0.063)	0.018
male	fT4	28	1.750 (0.954 - 2.546)	0.843 (0.460 - 1.226)	<.001
male	fT3	28	0.752 (0.372 - 1.131)	0.819 (0.406 - 1.233)	<.001
male	TSH	28	-0.016 (-0.032 - 0.001)	-0.443 (-0.902 - 0.016)	0.059
**Beta-blocker**					
no	fT4	91	0.979 (0.629 - 1.329)	0.859 (0.551 - 1.166)	<.001
no	fT3	91	0.482 (0.284 - 0.680)	0.787 (0.464 - 1.111)	<.001
no	TSH	91	-0.024 (-0.046 - -0.001)	-0.219 (-0.425 - -0.013)	0.037
yes	fT4	48	1.580 (1.129 - 2.031)	0.768 (0.549 - 0.987)	<.001
yes	fT3	48	0.757 (0.490 - 1.025)	0.799 (0.517 - 1.080)	<.001
yes	TSH	48	-0.048 (-0.110 - 0.013)	-0.409 (-0.929 - 0.111)	0.123
**Thyrotoxicosis**					
yes	fT4	61	1.141 (0.645 - 1.638)	0.611 (0.345 - 0.876)	<.001
yes	fT3	61	0.608 (0.337 - 0.880)	0.662 (0.367 - 0.957)	<.001
yes	TSH	61	-0.000 (-0.000 - 0.000)	-0.038 (-0.158 - 0.082)	0.511
no	fT4	78	0.106 (-0.058 - 0.271)	0.199 (-0.109 - 0.508)	0.206
no	fT3	78	0.010 (-0.025 - 0.046)	0.076 (-0.184 - 0.336)	0.569
no	TSH	78	-0.034 (-0.099 - 0.030)	-0.173 (-0.498 - 0.153)	0.298
**Center**					
St. John	fT4	70	1.212 (0.867 - 1.557)	0.988 (0.707 - 1.269)	<.001
St. John	fT3	70	0.579 (0.415 - 0.743)	0.934 (0.669 - 1.198)	<.001
St. John	TSH	70	0.001 (-0.011 - 0.014)	0.026 (-0.199 - 0.252)	0.819
MUG	fT4	69	1.680 (1.183 - 2.178)	0.796 (0.560 - 1.031)	<.001
MUG	fT3	69	0.793 (0.525 - 1.062)	0.796 (0.527 - 1.066)	<.001
MUG	TSH	69	-0.062 (-0.113 - -0.012)	-0.431 (-0.780 - -0.082)	0.016

Abbreviations: fT4, free thyroxine; fT3, free triiodothyronine; TSH, thyroid-stimulating hormone; RHR, resting heart rate; MUG, Medical University of Graz

Table S3. Stratified analyses of binary logistic model generalized estimating equation (GEE) analyses for associations between thyrotoxicosis and RHR-Fitbit-10d.

Category	n	Odds ratio (95% CI)	Odds ratio per SD HR increase (95% CI)	P-value
female	111	1.404 (1.215 - 1.622)	17.392 (5.141 - 58.839)	<.001
male	28	1.188 (1.022 - 1.380)	2.657 (1.134 - 6.225)	0.024
no	91	1.210 (1.030 - 1.420)	4.375 (1.257 - 15.223)	0.020
yes	48	1.203 (1.091 - 1.326)	6.830 (2.465 - 18.920)	<.001
St. John	70	1.126 (1.036 - 1.224)	2.886 (1.372 - 6.071)	0.005
MUG	69	1.546 (1.240 - 1.926)	43.287 (6.442 - 290.862)	<.001

Thyrotoxicosis is defined as FT3 (free thyroxine) and/or FT4 (free triiodothyronine) above the reference range

Abbreviations: RHR, resting heart rate; MUG, Medical University of Graz

Table S4. Linear model generalized estimating equation (GEE) analyses for associations between thyroid function tests and RHR-Fitbit-10d when beta-blocker users have their RHR increased by 11 bpm.

Hormone	n	Unstandardized b (95% CI)	Standardized beta (95% CI)	P-value
fT4	139	1.169 (0.861 - 1.478)	0.837 (0.616 - 1.058)	<.001
fT3	139	0.551 (0.405 - 0.698)	0.820 (0.602 - 1.039)	<.001
TSH	139	-0.024 (-0.045 - - 0.004)	-0.263 (-0.481 - - 0.044)	0.019

Abbreviations: fT4, free thyroxine; fT3, free triiodothyronine; TSH, thyroid-stimulating hormone; RHR, resting heart rate

Table S5. Binary logistic model generalized estimating equation (GEE) analyses for associations between thyrotoxicosis and RHR-Fitbit-10d, when beta-blocker users have their RHR increased by 11 bpm.

n	Odds ratio (95% CI)	Odds ratio per SD HR increase (95% CI)	P-value
139	1.178 (1.075 - 1.291)	5.798 (2.178 - 15.437)	<.001

Thyrotoxicosis is defined as fT3 and/or fT4 above the reference range

Abbreviations: RHR, resting heart rate

Table S6: Beta-blocker medication and daily dosage per study participant and study visit

Participant	Visit 1	Visit 2	Visit 3	Visit 4	Visit 5	Visit 6	Visit 7
1	–	–	–	–	–	–	–
2	Inderal, 60 mg						
3	Beloc, 25 mg	–	–	–	–	–	–
4	–	–	–	–	–	–	–
5	Bisoprolol, 2.5 mg	Bisoprolol, 2.5 mg	Bisoprolol, 2.5 mg	Bisoprolol, 2.5 mg	Bisoprolol, 2.5 mg	Bisoprolol, 2.5 mg	Bisoprolol, 2.5 mg
6	Nomexor, 5 mg	Nomexor, 5 mg	Nomexor, 5 mg	Nomexor, 5 mg	Nomexor, 5 mg	Nomexor, 5 mg	Nomexor, 5 mg
7	–	–					
8	Inderal, 40 mg	Inderal, 40 mg	Inderal, 40 mg	Inderal, 20 mg	Inderal, 20 mg	Inderal, 20 mg	
9	–	–	–	–	Inderal, 40 mg	–	–
10	–	–	–	–	–	–	–
11	–	–	–	–	–	–	
12	–	–	–				
13	–	–	–	–	–	–	
14	–	–	–	–	–	–	–
15	–	–	–	–	–	–	
16	–	Inderal, 60 mg	Inderal, 120 mg	Inderal, 40 mg	Inderal, 40 mg	–	–
17	–	–	–	–	–	–	
18	Inderal, 120 mg	Inderal, 120 mg	Inderal, 120 mg	Inderal, 120 mg			
19	–	–	–	–	–	–	–
20	–	–	–	–	–	–	–
21	–	–	–	–	–	–	
22	–	Inderal, 60 mg	Inderal, 60 mg	Inderal, 20 mg	Inderal, 20 mg	Inderal, 60 mg	Inderal, 60 mg
23	–	Inderal, 30 mg	–	–			
24	–	Inderal, 80 mg	Inderal, 40 mg	–	–	–	–
25	Bisoprolol, 2.5 mg	Bisoprolol, 2.5 mg	Bisoprolol, 2.5 mg	Bisoprolol, 2.5 mg	Bisoprolol, 2.5 mg	Bisoprolol, 2.5 mg	–
26	Inderal, 10 mg						
27	–	–	–	–	–	–	
28	–	Inderal, 20 mg	–	–	–	–	–
29	Bisoprolol, 5 mg	Inderal, 80 mg	Inderal, 60 mg	Inderal, 20 mg	Inderal, 20 mg	Inderal, 20 mg	–
30	–	Inderal, 60 mg	Inderal, 60 mg	Inderal, 60 mg	Inderal, 20 mg	Inderal, 20 mg	Inderal, 60 mg

Drug name (active ingredient): Inderal (propranolol); Beloc (metoprolol); Bisoprolol (bisoprolol); Nomexor (nebivolol).
